# Hysteria1Read at the twenty-ninth annual meeting of the Medical Association of Central New York, October 20. 1896.

**Published:** 1897-02

**Authors:** B. C. Loveland

**Affiliations:** Clifton Springs, N. Y.


### HYSTERIA.[1]

By B. C. LOVELAND, M. D., Clifton Springs, N. Y.

THE subject of this paper occupies about the same proportion of the time of the specialist in nervous diseases as the various forms of indigestion do with the general practitioner. And there is not a general practitioner of any experience who has not one or more cases on his hands, which have come to be his particular "thorn in the flesh," or, failing to receive the benefit they or their friends think they should, have stopped calling for his ministrations, and fallen to talking over among their neighbors the story of their distressing illness, how this and that and the other doctor have tried all their skill on them in vain, and in fact take a melancholy pleasure in the reputation of being the "sickest and most patient under unremitting suffering of all in the neighborhood."

To neglect the subject or treat it lightly is to ignore a matter which affects nearly every family among our patients, and often the physician's family is not exempt, while not a few who have treated others, themselves become the victims of the malady. The distribution of this disorder is not limited by lines of nationality, of age or of sex. It appears alike among young and old, male and female. We commonly regard it as a disease, but it is often met with in what we call health. In looking for a definition of hysteria we are met by numerous descriptions of its phenomena, but a definition at once concise and accurate is hard to find. One of the best is from Wood's Atlas of the Nervous System, by Dr. Jakob, and is as follows: "Hysteria is a psychosis, characterised by pathologic disturbance of mental conception and the will, which leads to an inexhaustibly large number of functional anomalies of the motor and sensory parts of the body, for the explanation of which no organic lesion can be found." If I were to define hysteria, I would say it is a condition in which the person's judgment and will, regarding himself or herself, is dominated by the emotional nature and not by the reason. And the object of this study is to arrive at a better conception of the workings of the hysterical mind, so that we may be able to more successfully meet the conditions, and also be on the alert to prevent the development of the condition in the families where we are called on as medical advisers.

1. Read at the twenty-ninth annual meeting of the Medical Association of Central New York, October 20, 1896.

If I may say a word as to the diagnosis of hysteria, it will be this: hysteria cannot be correctly diagnosticated without a sufficiently broad knowledge to differentiate it from all the diseases it may simulate. It is mortifying to the physician and disastrous to the patient when strychnia poisoning, for instance, is mistaken for hysteria, but this mistake has been made. And I might mention others. I do not believe that the hysterical patient is so from choice, or that the feeling about this disorder, commonly held among the laity,—and, I am sorry to say among many physicians also,—that hysteria is a synonym for obstinacy has any adequate foundation in fact. In the earlier manifestations, neither the patient nor those who care for the patient appreciate the real situation, and the patient is either treated as a *very* sick person, the whole household turning in to help in relieving her misery, thereby educating her to believe she *is* very sick; or the other extreme is applied, and the patient is made to feel that her friends believe her to be shamming, in other words a hypocrite. This treatment usually is sufficient to develop any amount of perverseness. By what I have just said I do not mean to infer that shamming never occurs in hysteria, for it does; but when it does it is the result of circumstances which seem to the patient to fully justify the deception. For instance, a young woman who has had every attention, whose illness has been somewhat protracted, doctor after doctor has been called, each looked wise, said little, and gave her something which she was led to believe would give her permanent relief. Perhaps relief followed for the time, but soon her old symptoms all return. She loses all faith in doctors and medicines. Each doctor said he knew what her trouble was, and promised her relief, but all failed to remove her bad feelings, or to refer them to the proper source. Is it any wonder that after such an education the poor creature has come to think in part at least with her numerous friends and advisers, that there must be some real disease at work in her, or else she could not feel so badly as she does? Now comes a change in affairs. She has gotten beyond her own unaided power to control, and beyond the power of her friends to endure.

They go to their family doctor, or more likely at this stage take her to a specialist, describe the lamentable condition into which she has drifted, and beg him, if possible, to do something to help the sufferer. The doctor then tells the truth, which before he has either withheld or been unaware of. The attitude of her friends toward her changes, she gets no more sympathy, is told that nothing is the matter with her, and that she could do differently if she wanted to, and other things equally soothing. Is it any wonder then that, believing as she does, she tries to convince her friends of the genuineness of her suffering? The effort to do this leads to minute descriptions of her pains or other feelings, which requires a very careful analysis, and more thought than ever on them. Then, if the pushing and opposition are kept up in the same spirit, of course it will be met by bad temper, and we will see the development of the churlishness usually thought to be inseparable from the malady. An illustration of the kind of education which terminates in hysteria, came under my observation a short time ago. An over-anxious mother and her four-year-old daughter were under my care. The little girl while at play falls down and hurts herself. She comes to her mother complaining of her hand. The mother gives the hand a good deal of attention, often asking if the hand still hurts. The child has it rubbed, and still whines about it. The mother can see nothing wrong, but thinks it possible that a bone is broken, and sends for the doctor. Nothing wrong, whatever, can he discovered, not even the place where the hand was struck. Advice is given not to pay any attention to it, and the mother says, "Why not put a bandage on it? My children seem to derive the greatest comfort from a bandage. In fact, they have always some of them got bandages on at home." And the hand is bandaged, bandage coming off while the child is asleep, and the hand is forgotten in the morning and therefore well. Partly through heredity, partly through education, this child of four years shows distinct signs of hysteria, but at her age I believe it could be entirely cultivated out. Just a word concerning the relation between hysteria and neurasthenia. I do not think the case of neurasthenia exists, in a chronic form at least, without more or less hysteria.

Some months ago there appeared in the *Medical Record*, an editorial, which quoted at length from an address by Professor Erb, of Heidelberg, which was in substance that the whole tendency of the times, the mad rush for success early in life, for the securing of a fortune at a time in life when our fathers would have been abundantly satisfied with a competency, the habit of taking a trip of sightseeing when overworked, instead of taking a needed vacation for rest, helped on as they are by our systems of telegraph, telephone and rapid transit on sea and land, all these point to a morbid tendency of the times. And as a result of this state of affairs, says the learned Professor, "we have the neurasthenic, a refined expression of hysteria and hypochondriasis." In other words, he treats the words as synonymous and the conditions as phases of the same thing. From Dr. H. C. Wood I quote : "Neurasthenia is not a disease, but a condition of weak nerves, a root on which is often grafted hysteria, hypochondriasis, melancholia and insanity." A large proportion of the cases I see diagnosticated as neurasthenia are, I am sure, cases of chronic hysteria. Today I see a man, sound in physique, active in mind, absorbed in business, accounted not only a healthy man, but an unusually bright business mind. He meets with an accident, breaks his arm, is laid up from going to the office for two or three weeks. His arm gets well, but he has nervous prostration, is unable to attend to his business, and consults a nervous specialist who tells him he is suffering from neurasthenia, weak nerves, the result of overtaxation and ushered in by the shock to his nervous system which attended the accident. How often is this picture repeated ? The patient is told that there is nothing to do but to take a longer vacation, and perhaps is put on a rest cure. After more or less time spent in this way the patient begins to wonder why he does not regain his strength as his advisors said he would, and consults other and perhaps more eminent physicians. They look him over, examine him thoroughly, find every organ sound, and begin to wonder if his mind is all right ; finally concluding that he is simply a victim of hysteria, they tell him that he has no disease, that he needs occupation, that he can take up work if he would only stop thinking of his feelings, and the like. Now it is as difficult to make him believe this, which is the truth, as it would have been six months or a year ago to have convinced him that he was then doing the best thing he could to bring about the condition he is now in, which is equally true.

The point I would make is this. In the case referred to the man was essentially hysterical when he was called well, a bright, good business man, and so forth. The only difference being that then his judgment was overridden or controlled by his ambition, or his passion for riches or fame, to such an extent that he was constantly working more hours than his reason would approve ; while now his ambition, or passion for gain or glory, has been displaced in its control of his judgment by his bodily feelings, another and lower order of his emotional faculties. In neither state is his judgment directed by his reason, which, it is needless to say, is the only guide for the judgment. This is the condition which I call hysteria, whether it be found in what we term health or disease, and it is, as I have indicated in the foregoing illustration, only the chance of an accident, apparently slight, when one is in this condition, which marks the difference between the person termed well, active, bright, and so on, and the one usually termed a "crank," a fraud, imposing on patient friends. Hence the importance of appreciating the condition whether in health so-called or in disease.

I do not mean to go into the treatment of hysteria in detail, but will state a few facts which will commend themselves to everyone who has had any experience in handling these unfortunate cases. First, the patient needs to be removed from the influence of her family. This seems unnecessary and often absurd to the family or friends of the patient, but the sympathy and attention of the family is as inimical to the recovery of the hysterical patient as it would be to have some one keeping up a daily supply of liquor for a patient you were trying to break of the alcohol habit. Second, all the attention should be paid to establishing such habits of eating, drinking and exercise as will tend to increase her health to the highest possible point. Third, pay as little attention as possible to the symptoms as they arise, except to keep up a strong moral treatment, encouraging her to disregard her feelings, and divert herself with anything to employ her hands, explaining to her what hysteria means and how it is conquered. I have said nothing about the medical treatment of hysteria thus far, because I think it is so much less important than the moral treatment. It may be necessary, in fact is often necessary, in advanced cases to give medicine at times, but anything she has come to think does her special good, has been used long enough, and should be dropped or changed immediately if we are to consider our patient's best good. For it will be remembered that we are trying to break a habit of the domination of the patient by the emotional faculties, which is marked by great dependence on outside helps, such as sympathy or encouragement of friends, medicines, stimulants, smelling salts, hot water bags, and often a number of other things. Now, if the habit referred to is to be broken, a habit of independence and self-forgetfulness must be cultivated. And this cannot be done so long as the patient is allowed to have either friends to sympathise, or medicines, or other remedies to turn to for every symptom, either real or imaginary. The development of hysteria is an educational process, and its cure is also an educational or culture cure.

At the close of this paper I wish to reiterate what I have already alluded to, that is, the importance that attaches to the physician's knowledge and advice in recognising and checking these unfortunate conditions in their incipient stage, and in preventing their development altogether, so far as his influence may go in the education of the children and young people under his care. This is said to be the age of preventive medicine, and much time and labor are devoted each year in that direction, but here is a field poorly worked, which I think will yield as rich a harvest as any, and might be made a great blessing to the oncoming generation, if not to the chronic hysterical sufferer.

